# "When My Information Changes, I Alter My Conclusions." What Can We Learn From the Failures to Adaptively Respond to the SARS-CoV-2 Pandemic and the Under Preparedness of Health Systems to Manage COVID-19?

**DOI:** 10.34172/ijhpm.2020.240

**Published:** 2020-11-29

**Authors:** Elisabeth Paul, Garrett W. Brown, Andreas Kalk, Wim Van Damme, Valéry Ridde, Joachim Sturmberg

**Affiliations:** ^1^School of Public Health, Université libre de Bruxelles, Brussels, Belgium.; ^2^Global Health Theme, University of Leeds, Leeds, UK.; ^3^Deutsche Gesellschaft für Internationale Zusammenarbeit, Kinshasa Country Office, Kinshasa, Democratic Republic of the Congo.; ^4^Department of Public Health, Institute of Tropical Medicine, Antwerp, Belgium.; ^5^CEPED, Institute for Research on Sustainable Development (IRD), IRD-Université de Paris, Paris, France.; ^6^School of Medicine and Public Health, Faculty of Health and Medicine, University of Newcastle, Callaghan NSW, Australia.

## Introduction

 Countries around the world have been hit by the severe acute respiratory syndrome coronavirus 2/coronavirus disease 2019 (SARS-CoV-2/COVID-19) pandemic, and have reacted to its spread in very different ways. Some countries (eg, Sweden) have implemented minimalist public health interventions, while others (eg, France, New Zealand) have imposed almost complete population lock-downs and/or other restrictions to freedom of movement and privacy.^1^ Such extreme interventions have complex impacts and come at a tremendous (mental) health and societal cost, disproportionately affecting the lowest socio-economic strata, and populations in low-and middle-income countries.^2-5^ However, the patterns of infection and mortality rates are extremely heterogeneous within and across individuals and countries.^6,7^

 Complex problems are not solved by universal (untargeted) interventions, as these rarely result in significant change for target populations, and typically perpetuate existing health inequalities.^8^ Policy-makers always have to consider trade-offs amongst intended (positive) outcomes versus the costs and possible unintended (negative) consequences.^9,10^ Therefore, “strategizing” health policies entails a solid situational analysis and an inclusive, transparent policy dialogue with appropriate participation, to prioritize interventions according to relevant criteria, including the vulnerability of health problems, making sure they focus on the most cost-effective interventions and are proportional to the burden of the problem.^11^ Public health policies also require openness, transparency, consistency of messaging, opportunity to appeal decisions so as to achieve public confidence, and avoid distrust, paternalism, or anxiety in accordance with the “accountability for reasonableness framework.”^12,13^ Inclusiveness and multidisciplinarity are even more necessary since the COVID-19 crisis is not simply a health problem but a societal one, impacting every single person in society one way or another.^14^

 We contend that the COVID-19 policy discourse across countries and continents has largely failed as they neglected these basic public health principles. We argue that the rapid growth in our understandings of SARS-CoV-2/COVID-19 dynamics necessitates the regular adaptation of public health policies and its transparent messaging to the general public, high risk populations and health professionals.

 This article is based on a collaboration of clinicians and experts in global public health and policy. We scanned the most relevant literature to provide a narrative review of the emerging evidence about SARS-CoV-2 and COVID-19. We outline ways to better integrate the rapidly emerging – at times seemingly contradictory – knowledge into more effective public health policies, and emphasise the need to more appropriately and effectively communicate them to various constituencies.

## Our Information About SARS-CoV-2 and COVID-19 Is Rapidly Changing, Calling for Policy Reflections

 Knowledge about COVID-19 (the disease) and its cause (the novel coronavirus SARS-CoV-2) is rapidly emerging, challenging effective decision-making in a context of uncertainty. We see marked heterogeneity in disease epidemiology and disease behaviour at the macro-level, with a relatively high level of SARS-CoV-2 sero-positivity in some settings. We also see heterogeneity in the biology of the virus and its effects on our immune responses at the micro-level, and their macro-level effects on transmission, pathogenicity and susceptibility remain poorly understood.^15-18^ At the patient-level COVID-19 exhibits highly variable symptoms – respiratory, neurological, gastrointestinal – that continue to puzzle clinicians.^7,19,20^ A complex picture is emerging where a majority of people infected remain asymptomatic, and where most symptomatic people have relatively mild disease.

 The epidemiology of SARS-CoV-2, and thereby its *infectiousness*, *case-fatality *and *infection fatality rates* remain highly contested. Compared to other pandemics, one would have to regard SARS-CoV-2 as moderately infectious, much less than measles, but more than influenza. As for its lethality, a model framework estimated a population infection fatality rates of 0.79% (with notable heterogeneity)^21^ and a recent meta-analysis found a median corrected COVID-19 infection fatality rate of 0.23%^22^ – against an infection fatality rate of 0.1%-0.2% for influenza.^23^ Measles is a good illustration of the fact that infection fatality rates are not a constant variable of a disease, but depend on contextual factors and social determinants, and evolve over time.^24^ This is also true of SARS-CoV-2 – the elderly, people with pre-existing conditions, and people with high viral load exposure are at highest risk, so that infection fatality rates vary considerably between settings and by age, with consistently low infection fatality rates for people below 65/70 years, but high variations – and far higher lethality – for older patients.^21,22^ However, estimated case fatality rates – as now suggested by second wave figures^25^ – and possibly also infection fatality rates, are decreasing, over time, not only because we now have better estimations of the denominator, but also because of a decreased “infection density” resulting in a lower probability to catch the virus and develop (severe) disease.^26^ Given the difficulties of accurate attribution, it has been suggested that ‘excess mortality’ provides a better estimate of the lethality of the SARS-CoV-2 outbreak and its variability across countries (see the EuroMomo tool for European countries: https://www.euromomo.eu/graphs-and-maps#excess-mortality). In light of the observed high degrees of variability, policy responses must be commensurate to emerging knowledge: a lower fatality rate than initially feared, and signs of diminishing risk of dying, leads to a very different appreciation when seen in the context of other health risks, and thus tempers the expected benefits of many current measures aimed at stopping the pandemic or even – as elusive as it may be – the eradication of the virus. For instance, even Belgium, where the fatality rate of COVID-19 is among the highest, the monthly fatality rate was lower than during other health emergencies of the last century.^27^

## SARS-CoV-2 Is Necessary but Not Sufficient to Cause COVID-19

 The SARS-CoV-2 pandemic results in a patterned outcome of COVID-19 disease: only a small number develop serious disease associated with high mortality, primarily the elderly and those affected by multimorbidity.^6,7,28,29^ Beyond comorbidities, like for all health problems, social determinants are emerging as an important contributor in the severity of COVID-19.^28^ This means that the relationship between the exposure to the virus (SARS-CoV-2) and the development of the disease (COVID-19) – and ultimately its outcomes – is not ‘binary,’ but is considerably influenced by additional factors. Put as an equation, instead of a causal linear relationship [SARS-CoV-2 => COVID-19], COVID-19 more realistically is modelled as a nonlinear relationship [(SARS-CoV-2 * X- * Y- * Z-Factors) => COVID-19]. A different way to put this is through Rothman’s lens of *sufficient causes* – while SARS-CoV-2 is necessary, on its own it is not sufficient to cause COVID-19.

 The multi-dimensional factors resulting in COVID-19 mean it is – in many cases – difficult to attribute COVID-19 as the *cause of death* rather than an *association with death*. The distinction is rarely made or transparently reported. Many jurisdictions count any *death associated with a positive SARS-CoV-2 swab* as a COVID-19 death, which overstates the *true fatality rate*.^30^ According to excess death counts, other models underestimate the true toll of COVID-19 and/or response measures presenting decision-makers with contradictory informantion.^27,31^ A differentiated understanding of the pandemic and its consequences necessitates the distinction between three states: (*i*) exposure to the virus; (*ii*) infection with the virus; and (*iii*) affected by COVID-19, the disease. We argue that it is of critical importance for policy-making to make this difference because if ‘X-, Y-, Z-factors’ are confirmed to be important causes of the ‘transition’ from infection to disease, these could, and should, be targeted or at least recognised as a crucial aspect of long-term disease control policies.

 COVID-19 reiterates the well-known relationship between comorbidities (eg, obesity and diabetes^28,29^), lifestyle factors (eg, low levels of vitamin D^32^) and low socioeconomic status on morbidity and mortality. In the context of a sustainable response strategy, policy should take these factors into account as a means to enhance broader population health benefits.^33^

 In other words, our policy thinking about COVID-19 must embrace a holistic *systems-in-systems approach* to public health in order to generate more sustainable, effective and equitable outcomes.^34^ This is not to suggest that immediate protective countermeasures are unnecessary, in some cases they are crucial, especially for the highly vulnerable. However, given what we know of this disease (and others), the current *ad hoc* and *reactive control tactics* often employed have shown mixed results (see for instance https://ourworldindata.org/coronavirus) and will remain sub-optimal if COVID-19 becomes endemic or if a safe, effective, and acceptable vaccine fails to emerge.

## Public Health Policies Need to Be Adapted Accordingly

 The phrase ‘*when my information changes, I alter my conclusions … What do you do, sir?*’ is attributed to John Maynard Keynes. As the pandemic progresses, together with our knowledge about it, public health policies unavoidably have to adapt, and their rationale needs to be communicated transparently so that it can be more readily accepted by the public and health professionals.^12^ For example, the initial strategy of ‘*flatten the curve*’ (‘Save the NHS’ in the United Kingdom) was designed to help health systems cope with the anticipated surge in caseload, and to guarantee optimal in-hospital care, as a way to minimise fatalities. Its underpinning rationale was straightforward and clear to a population that readily cooperated. This clarity unquestionably gained the public’s trust and support. However, in many countries, subsequent policies were not adapted to emerging knowledge, resulting in confusing and at times conflicting messages.

 For example, the constant focus on *absolute* numbers of ‘confirmed cases’ and ‘registered deaths,’ without providing evidence on the non-registered cases or attributable deaths, nor *providing a denominator* (eg, population, cohort tested, age groups), renders many figures meaningless and potentially misleading. Paradoxically, rather than effectively adopting public health messages to our growing understandings of the pandemic, some governments suddenly switched their focus to ‘restarting the economy.’ This muddling of the public discourse has resulted in the public’s loss of trust and confidence in policies and decision-makers. Equally, measures such as the hyped-up pronouncements of SARS-CoV-2 vaccines and antivirals, as well as the imposition of undifferentiated restrictions and obligations, seem to pursue (the overall aim rarely being disclosed) an illusionary goal of ‘eradicating’ the virus rather than preventing the disease in the now known high risk groups of the elderly (especially nursing home residents), those with other pre-existing conditions, and highly exposed health professionals.

 The emergence of a second wave of the SARS-CoV-2 pandemic in some countries coinciding with the realisation of the socio-economic consequences of the earlier lockdown has brought to the forefront the lack of well-thought out *systemic policy positions*. Control measures have been repeatedly tightened (eg, local lockdowns, quarantine after returning from travel abroad, school closures, wearing face masks in outside settings) at times when there only were a limited number of sick people, and despite the fact of insufficient evidence to support such measures.^35,36^ Measures adopted in several countries are probably not proportional to the severity of the threat,^37^ and could result in greater loss in life expectancy than would have been observed in their absence.^10^ Untargeted measures not only risk poor efficacy, but also perpetuate health-destroying impacts of socio-economic distress, place undue pressure on vulnerable populations, increase inequities, and are unsustainable. By contrast, the controversial ‘light-touch’ Swedish model has seemingly proved to be more sustainable in the medium term, but also highlights that decision-making in the realm of uncertainty inevitably entails undesired outcomes, as evidenced by the early failure to recognise the vulnerability of nursing home residents.^38^ Managing uncertainty and complexity is inherent to a newly emerging pandemic. This calls for transparent and open deliberation with all stakeholders, acknowledging that emerging data is sparse and most likely flawed, that evidence providing certainty many never materialise, that paradoxical responses may emerge that are not easily or readily explainable, and that the implementation of pragmatic interventions, adapted to local settings, have the potential to provide important knowledge while waiting for controlled trial evidence to be produced.^39^

## Ways Forward

 Evidence-based policy states that the good governance of evidence requires transparency and conceptual clarity.^40^ This begins with determining and communicating what the ultimate goals and concerns are, and how they should be addressed.^41^ Setting ‘the’ overarching policy goal must be informed by a clear understanding about the difference between the properties of the *virus *and those of the *disease*. Current ‘virus suppression policy-settings’ seem to have been successful in the short term, and locally, in some – but not all – settings (such as Iceland, South Korea, Taiwan, New Zealand or China – see https://ourworldindata.org/coronavirus). However, they have been unable to control the pandemic globally – nor locally in more open settings – in a sustainable and equitably way. Indeed, the different policy approaches employed by individual countries have resulted in variable, largely unpredictable and often inequitable outcomes, coupled with enormous negative side effects.^2,3,5,10^ Despite their obvious and at times publicly acknowledged failings, decision-makers continue to enforce these policies arguing they are unavoidable until an efficient vaccine can be delivered ‘to end’ the viral threat. This ‘single-minded focus’ fails to appreciate the systemic nature of the pandemic, and the need to articulate an overarching – system-wide – goal to manage the interconnected and interdependent medical tasks of prevention, protection and treatment without exacerbating other health risks, while maintaining social cohesion and preventing economic collapse.

 Second, shifting towards a *systems-focused* overarching goal entails the need to change strategies. Infection control measures require different technical and communication strategies to those to protect vulnerable groups from being infected, or to limit the chances of ‘transition’ from infection to disease. Broad-based systemic public health approaches pay great attention to the wider determinants of disease^34^; in particular environmental factors as they are known to impact physiological dysregulation.^42,43^ A greater *understanding* of the ‘X-, Y-, Z-factors’ and their interdependencies leading to the ‘transition’ from infection to disease would not only be more effective, but also less costly (in terms of indirect effects) than undifferentiated universal policies.

 Lastly, given the mishandling of the pandemic by political and public policy leaders, there is now an urgent need for local and national leaders to regain the confidence of their communities – the perception of ‘trust’ is paramount to achieve acceptance of policies that significantly infringe personal and community freedoms for the benefit of the greater good.^44^ Hence, health policies need to be decided and explained based on a credible and consolidated evidence through an inclusive policy dialogue.^11^ However, few countries have set up such processes, which limits meaningful scientific debate in an environment dominated by an abundance of poor quality and/or insufficiently adapted research, competition between epistemic authorities,^35,45^ and the media, which can perpetuate myths in line with their respective ideological backers.

 Openness, transparency and efficiency require that as information changes and new knowledge emerges, policy responses must adapt accordingly. This entails that policy-makers must freely acknowledge past mistakes that have arisen due to the lack of information at the time decisions had to be made.^46,47^ It is an essential step to restore trust by the public, and at the same time breaks potential patterns of pathway dependency and imprinting, where decision-making becomes resistant to change.

## Conclusion

 Now that we are no longer in the acute emergency phase of the SARS-CoV-2/COVID-19 pandemic, and observe that the multisectoral crisis it caused lasts longer than anticipated, it is time to adopt a more systems thinking, strategic, long-term approach to resolving the crisis. In terms of correcting failed policies and addressing the foreseeable challenges, we suggest that public health authorities and global health leaders consider seven interdependent issues in future decision-making:

- Delineate between viral infection (SARS-CoV-2 positivity) and viral disease (COVID-19).
- Focus research on understanding the complexities between viral spread and disease development; identify which factors are responsible for the transition from infection to disease; identify the broader determinants of the pandemic; identify more clearly those at risk; identify what treatments can ameliorate disease severity and prevent mortality.
- Adopt broader policy goals aimed at optimising people’s health more generally (and not just control the virus); ensure that the enforced public health measures are proportional to the severity of the threat and mitigate their unintended consequences.
- Investigate how to expand – globally and locally – the implementation of appropriate preventive measures, treatments and public health interventions adapted to local contexts; support health professionals in their task under their local constraints.
- Better understand and attend to the needs of the most vulnerable populations; identify those vulnerabilities that are easily amenable to interventions; consider if policies adversely affect vulnerable populations; consider the balance of ‘cost-effectiveness’ in terms of impact on population health against the preservation of equity.
- Adapt policies and interventions in light of emerging knowledge (evidence-based policy-making); engage in transparent dialogues with all stakeholders in developing and implementing policy choices.
- Develop appropriate communication strategies that build public trust and support: transparently explain the chosen policy rationales and their evidence base; admit that there always will be indeterminacies and that emerging insights will result in timely adaptation of policies and interventions.

Ultimately only time will tell if people in given settings have cross-immunity through encounters with other coronaviruses,^17^ or have a more generalised ‘competence’ of their immune system, or if exposure to a reduced viral inoculum still induces some degree of immunity, with low risk of (severe) disease,^26^ or if lasting ‘herd immunity’ second to asymptomatic spread will emerge while vaccines remain under development, or if SARS-CoV-2 will just continue to come and go in waves as other coronaviruses do. At the moment, the answers to these questions remain open. So should our thinking: when information changes we should not be afraid to alter our conclusions. This is because when the facts and uncertainties are communicated effectively, with an open and transparent debate toward the collective end of better overall population health, it can help sharpen collective efforts while hopefully also reducing unnecessary anxiety and panic.

## Ethical issues

 Not applicable.

## Competing interests

 Authors declare that they have no competing interests.

## Authors’ contributions

 EP first conceived the paper, and after discussion with JS, wrote the first draft. All authors contributed equally to the development of the final version of the paper, and approved the final version.
